# Impact of COVID-19 Pandemic Lockdown on Mental Well-Being of Norwegian Adolescents During the First Wave—Socioeconomic Position and Gender Differences

**DOI:** 10.3389/fpubh.2021.717747

**Published:** 2021-09-14

**Authors:** Arnhild Myhr, Linn Renée Naper, Indira Samarawickrema, Renate K. Vesterbekkmo

**Affiliations:** ^1^SINTEF Digital, Steinkjer, Norway; ^2^Faculty of Health, University of Canberra, Canberra, ACT, Australia; ^3^Regional Drug and Alcohol Competence Centre, St. Olavs University Hospital, Trondheim, Norway

**Keywords:** mental well-being, adolescents, COVID-19, socioeconomic inequalities, Norway, lower secondary school

## Abstract

**Background:** The lockdowns associated with the COVID-19 pandemic has been called a crisis in mental health, and adolescents may have been among the most affected. Comparing the first period of societal lockdown in spring 2020 to periods going back to 2014 using a rich cross-sectional dataset based on repeated surveys, we explore the potential changes in self-reported mental well-being across sociodemographic groups among Norway's adolescents.

**Methods:** Norway closed schools and implemented strict restrictions in March 2020; an electronic questionnaire survey was distributed to lower secondary school students in Trøndelag county (*N* = 2,443) in May 2020. Results were compared with similar surveys conducted annually in the same county dating back to 2014. Logistic regression models were applied to investigate potential changes in depressive symptoms, loneliness, and quality of life and life satisfaction, and to detect possible differences in the impact of lockdown between the genders and socioeconomic groups.

**Results:** The prevalence of boys and girls reporting high quality of life (43–34%; 23–16%) and life satisfaction (91–80%; 82–69%) decreased significantly compared to the pre-pandemic. For girls only, lockdown was associated with higher odds for reporting high depressive symptoms. As expected, the least privileged socioeconomic groups showed the greatest psychological distress. However, our trend analyses provided no evidence that the socioeconomic inequalities in psychological distress (according to prevalence of high depressive symptoms or loneliness) changed substantial in any direction during the first wave of the pandemic [between the pre-pandemic and inter-pandemic periods].

**Conclusion:** Adolescents are vulnerable, and interventions should provide them with mental health support during crises such as societal lockdown. In particular, the social and health policy, public health, and further research should target these least privileged groups.

## Introduction

Like most countries, Norway closed its borders and introduced a nationwide-lockdown in March 2020, in order to slow the spread of COVID-19 and to lessen pressure on its healthcare system. This led to, among other things, closed schools, a temporary ban on leisure activities, and requirements for social distancing. Not since World War II has Norway's population been subjected to such drastic restrictions as during the first wave of the pandemic.

The abrupt and sudden change in everyday life was challenging. The adolescents and young people, who rely heavily on peer connections for emotional and social support, and for social development were particularly vulnerable (1, 2). Moreover, the links between interpersonal stress and the onset of emotional distress are strong among adolescents (3). Psychological stressors like financial insecurity, concern for one's own and others' health, lack of social and physical activities, and boredom, negatively impact the mood and mental well-being (4–7). A review of the studies on the impact of quarantines prior to 2020 by Brooks et al. (8) eight found that the psychological effects may be wide-ranging, substantial and long lasting, leading to more tension, irritability, and family conflicts. In addition, social isolation and loneliness have been associated with negative mental health outcomes (9); leading to the politicians and mental health advocates, and other professionals expressing concern about impacts of the COVID-19 lockdowns on adolescents' mental health and well-being, especially among those already in vulnerable life situations (10).

Findings from studies prior to the COVID-19 pandemic shows that the majority of Norwegian adolescents were satisfied with their lives. They had good relations with their parents, teachers, and friends. Also, they were satisfied with their schools and local communities and optimistic about their futures (11–13). Norwegian adolescents in low-income families have reported a lower life satisfaction than those in families with high socioeconomic positions (SEPs) (11, 14). A study on trends in socioeconomic inequalities in Norwegian adolescents' mental health from 2014 to 2018 reported higher symptom load with decreasing SEP levels for both boys and girls (15). In addition, as in many other high-income countries, Norway's adolescents, and young adults, especially girls, have shown an increasing trend of subjective mental-health complaints and loneliness in the past decade (11, 12, 16). In this trend the connection between SEP and self-reported psychological distress has persisted (11). The societal lockdown during the first wave of the pandemic unprecedently changed the young people's daily routine and how they organized themselves socially. Withdrawal from the school, social life, and leisure activities in addition to spending more time at home were among the most significant changes. A growing body of literature suggest that the experience of disruption from daily routine and social scaffold due to the school closures may increase the stress responses and pose a threat to mental health in adolescents (17, 18). Such impacts should be weighed against future decisions to close schools during pandemics. While the evidence from past epidemics suggest that closing schools can have a significant effect on reducing infection rates and flattening the curve (19, 20); recent modeling studies suggest that school closures have had far less impact than other social distancing interventions on the spread of COVID-19 (17).

Although several studies have investigated psychological effects of lockdowns on adolescents (21–32), only one have compared their mental state during lockdown with data collected before the pandemic and explored the impact of such across gender and socioeconomic subgroups (33), in spite of evidence that some groups seem to be more vulnerable to the mental health burden of the COVID-19 pandemic (34).We address these research gaps by using a rich cross-sectional dataset based on repeated surveys of adolescents in Norway begun in 2014 and repeated during the lockdown. The present study explores the following research questions:

1. Has mental well-being, defined as life satisfaction, quality of life, depressive symptoms, and loneliness, among adolescents attending lower secondary school changed during lockdown, compared to the pre-pandemic situation?
2. Are gender and family SEP related to adolescents' mental well-being and has this potential association changed from pre-pandemic to lockdown situations times?

## Materials and Methods

### Study Procedure

This study is based on data from two similar questionnaires administered in spring 2020: before lockdown (T1) and during lockdown (T2), as society reopened after the first wave of the COVID-19 pandemic. The questionnaire data collected prior to the pandemic is from the Norwegian national youth survey, Ungdata (12, 35, 36). Ungdata is an annual cross-sectional, quality assured and standardized system that surveys adolescents attending lower and higher secondary education in Norway (37). It is administered at school during the school hours, and the participation is voluntary, based on the parent's' informed consent. The survey covers a wide range of aspects of Norwegian youths' lives, and it is an important source of information on young people's health and well-being, both at the municipal and national levels. The Welfare Research Institute NOVA (at OsloMet) is, together with Norway's seven regional drug and alcohol competence centers (KoRus), responsible for conducting the survey. For a comprehensive description of the Ungdata survey, see www.ungdata.no/english/.

Based on the Ungdata questionnaire researchers at NOVA designed a similar COVID-19-relevant questionnaire and offered it to KoRus—Midt to use to survey the adolescents in Trøndelag county. All 38 municipalities in Trøndelag county were invited to take part in the COVID-19 survey, of which 10 participated. The survey took place between the 14th and 20th of May using open link access to ensure anonymity. Participation was voluntary and based on the parents' informed consent. As national restrictions gradually were relaxed beginning May 11, municipalities opened their schools at different times and at different paces. The location of the adolescents responding to the T1 questionnaire was the school classrooms and either the classrooms or at home in T2 due to prevailing COVID-19 conditions.

In addition to T1 and T2, supplement analyzes consist of respondents from the Ungdata survey in Trøndelag County during the period from 2014 to 2018. Due to a pre-planned extensive survey in 2020 Ungdata was not conducted in 2019. Norway's third largest city, Trondheim, is in this county, however, as they are considerably more populated and has only participated once during this time period they are not included in this study.

### Study Sample

Our study sample consists of students in the 10 participating municipalities enrolled in level 2 in the International Standard Classification of Education (ISCED). In Norway, students generally begin ISCED level 2 at age 13 and complete it the year they turn 16. Programs classified at ISCED level 2 may, for example, be referred to as “junior secondary school,” “middle school,” or “(junior) high school”; For international comparability we will use the term “lower secondary education,” as recommended by the ISCED. In this study, all students attending lower secondary education who either completed the Ungdata survey spring 2020 before the pandemic (T1, *n* = 2,443) or the COVID-19 survey lockdown (T2, *n* = 2,011) were included. We excluded individuals with missing information on gender (*n* = 77) and family SEP (*n* = 294). There was no missing information on school grade level. A total of 2,126 and 1,957 adolescents completed the surveys at T1 and T2, respectively. The percentage of boys and girls who completed the survey was evenly distributed between 8th (33 vs. 32%), 9th (32 vs. 33%), and 10th (35%) school grade level. The share of adolescents with a low family SEP was higher in the T1 sample compared with the T2 sample in both boys (21 vs. 12%) and girls (19 vs. 10%). The study sample was further reduced in the parametric estimations due to individuals missing information on depressive symptoms (*n* = 175), loneliness (*n* = 193), quality of life (*n* = 92), and life satisfaction (*n* = 76).

We apply supplementary trend analyses of depressive symptoms (*n* = 16,940) and loneliness (*n* = 16,847) across socioeconomic groups in the county of Trøndelag from 2014 until the lockdown (T2).

### Measures

#### Depressive Symptoms

Depressive symptoms were measured by means of six items derived from the Hopkins Symptom Checklist, constituting the “Depressive Mood Inventory” (38, 39). Adolescents reported if they during the past week had been affected by any of the following issues: “felt that everything is a struggle”; “had sleep problems”; “felt unhappy, sad, or depressed”; “felt hopeless about the future”; “felt stiff or tense”; and “worried too much about things.” Each item was answered on a four-point scale ranging from “not at all” (1) to “a great deal” (4).

A mean symptom score was constructed by adding up the scores (1–4) on all the items and dividing it by the number of completed items, given response to at least half of the statements. Furthermore, we constructed a dichotomous variable identifying adolescents reporting moderate to high depressive symptom load. Similar to (12, 13) we used a cutoff score of 3. Thus, we considered those adolescents who, on average, report at least “quite a lot of ailments” to have a high depressive symptom load.

#### Loneliness

Symptoms of loneliness were measured by asking the adolescents to rate, on a four-point scale ranging from “not at all” (1) to “a great deal” (4), whether they had “felt lonely” during the past week. We constructed a dichotomous variable identifying adolescents who reported that they had felt lonely “quite a lot” or “a great deal” during the past week.

#### Life Satisfaction

Life satisfaction was measured using the Cantril's ladder (40), which is a widespread measure (41, 42). The students were asked to rate satisfaction with their own lives on a scale from 0 (worst possible life) to 10 (best possible life). Similar to Samdal et al. (41) we apply a cutoff score of ≥6 to identify adolescents with high life satisfaction.

#### Quality of Life

Based on a report from the Norwegian Directorate of Health on measuring subjective quality of life (43), the 2020 surveys included six individual questions on positive emotions and experiences of mastery and meaning in own life. With answer categories from 1 (not at all) to 5 (all the time) adolescents wear asked to think about the past week and how often they had: “been happy”; “had lots of energy”; “been engaged”; “felt like you were mastering things”; “felt useful”; and “been optimistic about the future.” A mean score was constructed by adding up the scores (1–5) on all the items and dividing it by the number of completed items, given response to at least three of the statements. Adolescents who answered “often” or “all the time” were categorized as having high quality of life and contrasted with all other adolescents (i.e., a cut-off score of 4 for a dichotomous variable).

#### Socioeconomic Position

Ungdata surveys do not include questions about parents' occupations or incomes, largely to protect respondents' anonymity. However, they include a number of questions that relate to SEP (44). These include questions about their parents' educational level and the number of books at home as well as the four-point measuring instrument Family Affluence Scale II, which elicit number of cars, computers and/or tablets in the family, number of holiday trips in a year, and whether the adolescent has their own bedroom (45). A critical review of each question included in the collective affluence measure, as well as detailed information on how the measure is developed, appears in Bakken et al. (44). We calculated a mean sum score, ranging from 0 to 3, for each study participant. Thereafter n the total study sample was split into three equally sized groups ordered by increasing affluence level from low to high (low, medium, and high). Each of the dimensions used have some clear limitations as measures of a family's socioeconomic situation; as a collective index they probably provide a more robust and valid measure (46).The scale has been validated alongside other measures of adolescents' SEP and compared to measures in which adolescents report their parents' income, occupations, and education levels, and FAS II has better criterion validity and less susceptibility to non-response bias (47).

#### Covariates

Previous studies show that demographic variables such as gender and age may predict mental well-being in adolescents (15, 36, 48). We thus adjusted for gender and school grade level (proxy for age) in all our parametric models. School grade levels were categorized as follows: 8th grade (first year in lower secondary education starts normally at age 13), 9th grade, and 10th grade (last year in lower secondary education ends normally at age 16).

### Statistical Methods

**Figure 1:**
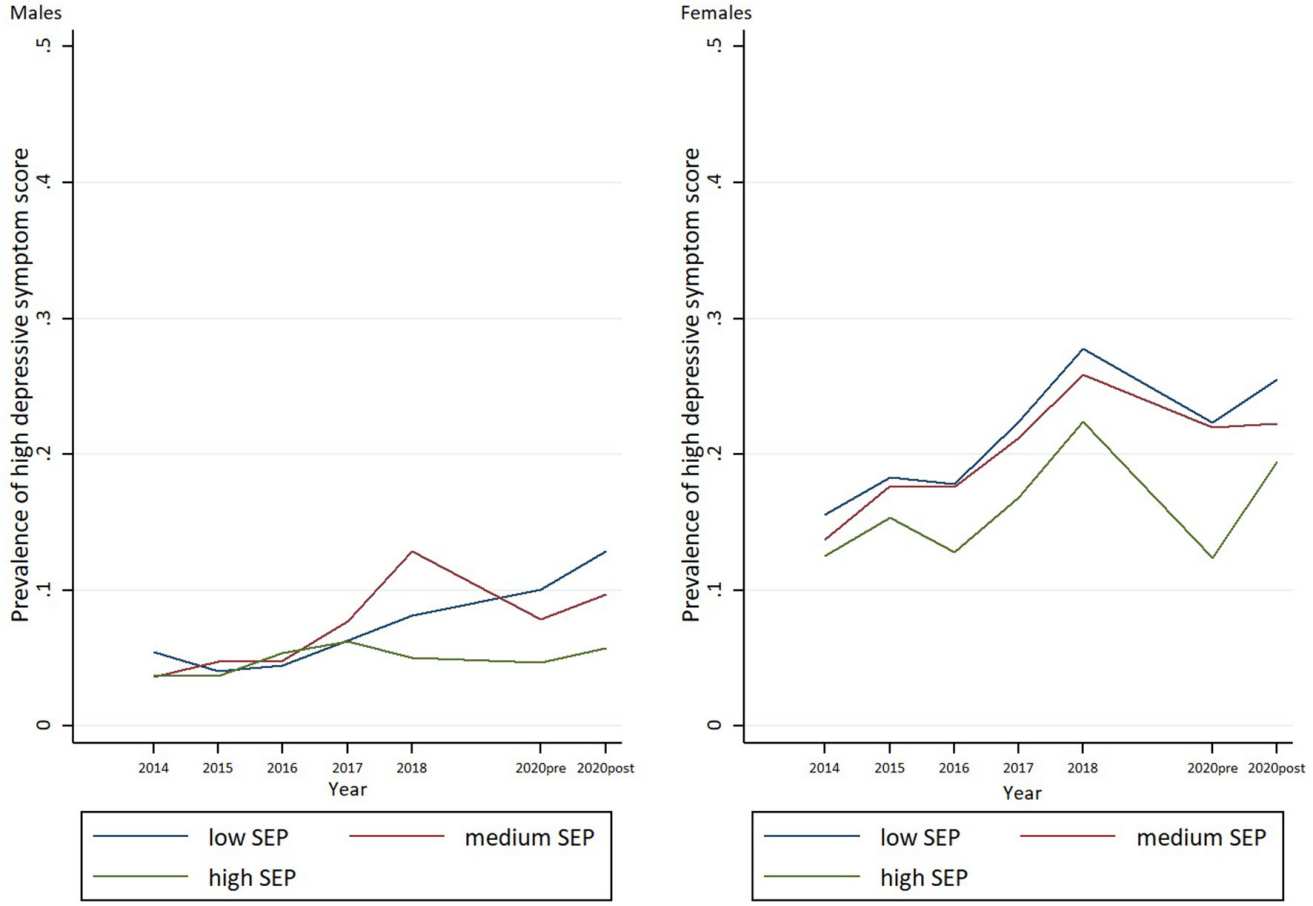
Trends in prevalence's of high depressive symptoms by SEP among boys and girls between 2014 and lockdown spring 2020.

**Figure 2:**
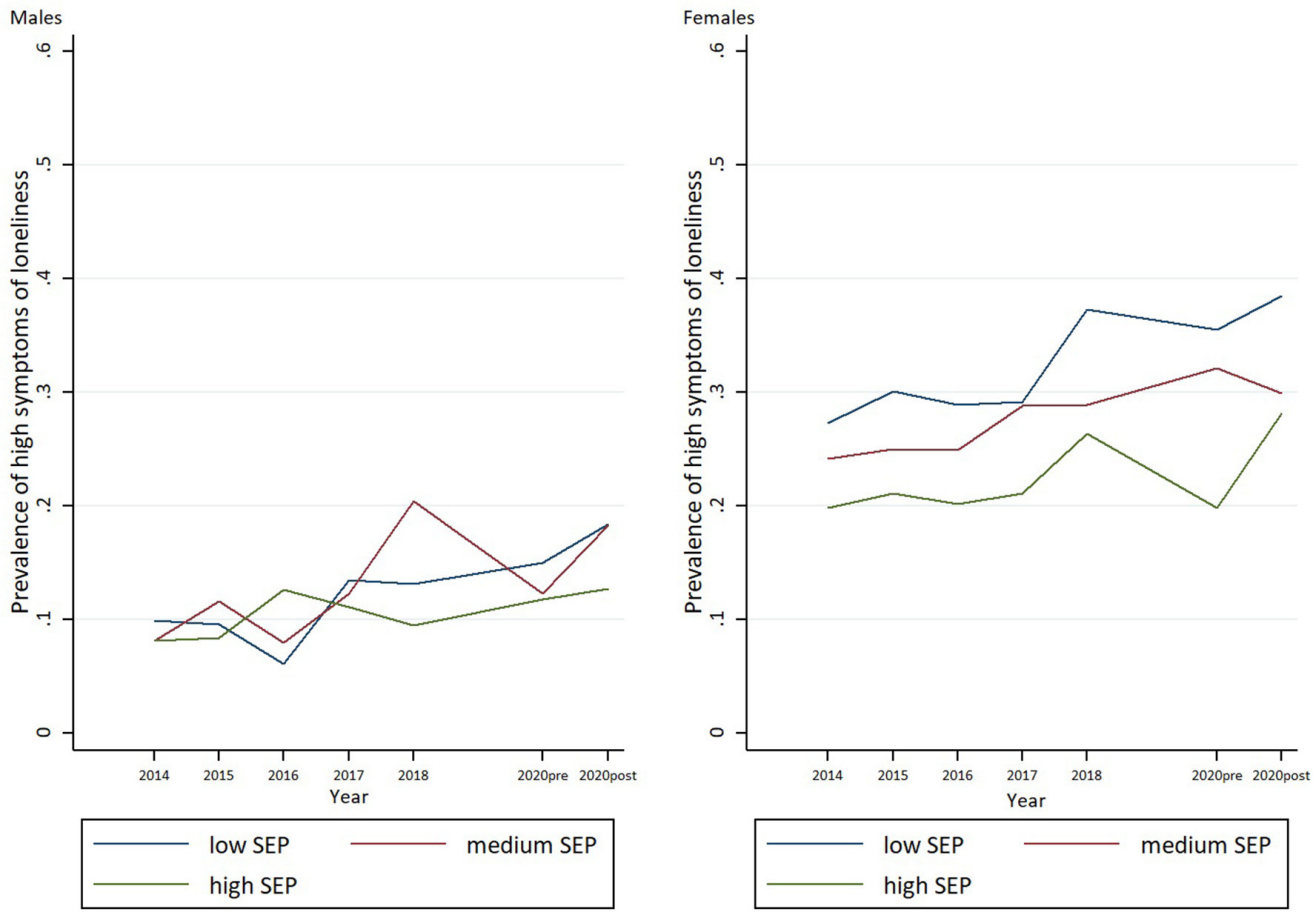
Trends in prevalence's of loneliness by SEP among boys and girls between 2014 and lockdown spring 2020.

First, descriptive analyses of percentages on demographic variables and the four mental well-being outcomes (i.e., depressive symptoms, loneliness, quality of life, and life satisfaction) were calculated, and the significance of the difference between T1 and T2 sample were tested by chi square tests (Table 1). Second, we examined changes in all four mental well-being outcomes following lockdown and potential inequalities between socioeconomic groups separately for boys (Table 2) and girls (Table 3) by using multiple logistic regression models (main effect models). The interaction term with SEP and lockdown was included to examine whether the potential SEP inequalities in adolescent's mental well-being have increased or decreased during lockdown. Third, we examined the associations between mental well-being and gender, and the hypothetical interaction with gender and lockdown (Supplementary Table 1). Fourth and finally, supplementary analysis of changes in depressive symptoms and loneliness across socioeconomic groups during the period from 2014 until lockdown was examined using logistic regression models (Figures 1, 2). We report odds ratios (OR) along with 95 % confidence intervals (95% CI). A threshold of 0.05 was used for statistical significance. All data management and statistical analysis were conducted in Stata/MP software (Version 13).

**Table 1:** Study characteristics and prevalence is of high level of depressive symptoms, loneliness, quality of life, and life satisfaction among boys and girls in lower secondary education in Trøndelag County, Norway—before and after the lockdown in spring 2020.

	**Boys (** ***n*** **=** **1,984)**				**Girls (** ***n*** **=** **2,099)**			
	**Pre-pandemic 2020 (** ***n*** **=** **1,020)**		**Lockdown 2020 (** ***n*** **=** **964)**		**Pre-pandemic 2020 (** ***n*** **=** **1,106)**		**Lockdown 2020 (** ***n*** **=** **993)**	
	**No**.	**%**	**No**.	**%**	**No**.	**%**	**No**.	**%**
**School grade level**
8th grade	339	33.2	295	30.6	354	32	352	35.4
9th grade	328	32.2	363	37.7	361	32.6	355	35.8
10th grade	353	34.6	306	31.7	391	35.4	286	28.8
**Family SEP**
Low	213^***^	20.9	115	11.9	210	19.0	96	9.7
Medium	360	35.3	386	40.0	359	32.5	378	38.1
High	447	43.8	463	48.0	537	48.6	519	52.3
**Prevalence's of high level** ^**a**^
Depressive symptoms	68	7.1	74	8.2	190	17.7	201	20.8
*Felt that everything is a struggle*	194	20.2	206	22.8	419^*^	39.0	418	43.6
*Had sleep problems*	182	18.9	193	21.4	292^**^	27.1	315	32.7
*Felt unhappy, sad, or depressed*	140	14.5	153	16.9	338^**^	31.6	364	37.7
*Felt hopeless about the future*	125^**^	13.1	161	18.0	282^*^	26.4	296	30.7
*Felt stiff or tense*	160	16.8	160	17.8	304^*^	28.6	315	33.2
*Worried too much about things*	231	24.1	193	21.5	538	50.2	480	50.3
Loneliness	123	12.8	141	15.6	289	27.0	285	29.7
Quality of life	440^***^	43.3	304	33.6	249^***^	22.6	155	16.0
Life-satisfaction	924^***^	91.1	739	80.1	901^***^	81.8	666	68.8

a *Comparisons between pre-pandemic and lockdown measures tested by Pearson chi square test*

* *p < 0.05*,

** *p < 0.01, and*

*** *p < 0.001*.

**Table 2:** The impact of family SEP and lockdown during COVID-19 pandemic^*^, and its interaction on the probability of high level of depressive symptoms, loneliness, quality of life, and life satisfaction among boys in lower secondary education.

**Boys**	**High depressive symptoms**				**Loneliness**				**Quality of life**				**Life satisfaction**			
	**Main effect**		**Interaction**		**Main effect**		**Interaction**		**Main effect**		**Interaction**		**Main effect**		**Interaction**	
**Predictors**	**OR**	**95% CI**	**OR**	**95% CI**	**OR**	**95% CI**	**OR**	**95% CI**	**OR**	**95% CI**	**OR**	**95% CI**	**OR**	**95% CI**	**OR**	**95% CI**
**School grade**																
8th grade	Ref		Ref		Ref		Ref		Ref		Ref		Ref		Ref	
9th grade	1.83	1.16–2.88	1.83	1.16–2.88	2.12	1.50–2.99	2.13	1.51–3.01	0.96	0.77–1.20	0.96	0.76–1.20	0.81	0.59–1.12	0.81	0.59–1.12
10th grade	1.61	1.01–1.01	1.61	1.01–2.57	1.74	1.22–2.49	1.76	1.23–2.52	0.89	0.70–1.11	0.89	0.70–1.11	0.96	0.69–1.33	0.96	0.69–1.34
**Lockdown COVID-19 pandemic**																
Pre pandemic 2020	Ref		Ref		Ref		Ref		Ref		Ref		Ref		Ref	
lockdown	1.23	0.87–1.75	1.28	0.62–2.63	1.27	0.98–1.66	1.24	0.67–2.32	0.65	0.54–0.79	0.51	0.31–0.84	0.37	0.28–0.49	0.47	0.27–0.85
**Family SEP**																
High	Ref		Ref		Ref		Ref		Ref		Ref		Ref		Ref	
Medium	1.70	1.14–2.53	1.67	0.92–3.04	1.26	0.94–1.69	1.00	0.47–1.28	0.95	0.78–1.17	0.88	0.67–1.17	0.69	0.52–0.93	0.74	0.44–1.25
Low	2.33	1.46–3.73	2.27	1.19–4.30	1.43	0.99–2.07	1.30	0.79–2.11	0.88	0.67–1.14	0.93	0.67–1.30	0.48	0.34–0.70	0.42	0.25–0.73
**Interactions SEP x lockdown**																
High^*^ lockdown			Ref				Ref				Ref		Ref		Ref	
Medium^*^ lockdown			1.03	0.46–2.30			1.50	0.83–2.71			1.16	0.78–1.75			0.91	0.49–1.72
Low^*^ lockdown			1.05	0.41–2.74			1.19	0.57–2.51			0.80	0.45–1.42			0.76	0.63–2.75

* *Adjusted for school grade*.

**Table 3:** The impact of family SEP and lockdown during COVID-19 pandemic^*^, and its interaction on the probability of high level of depressive symptoms, loneliness, quality of life, and life satisfaction among girls in lower secondary education.

**Girls**	**High depressive symptoms**				**Loneliness**				**Quality of life**				**Life satisfaction**			
	**Main effect**		**Interaction**		**Main effect**		**Interaction**		**Main effect**		**Interaction**		**Main effect**		**Interaction**	
**Predictors**	**OR**	**95% CI**	**OR**	**95% CI**	**OR**	**95% CI**	**OR**	**[95% CI**	**OR**	**95% CI**	**OR**	**95% CI**	**OR**	**95% CI**	**OR**	**95% CI**
**School grade**																
8th grade	Ref		Ref		Ref		Ref		Ref		Ref		Ref		Ref	
9th grade	1.80	1.35–2.39	1.77	1.33–2.36	1.77	1.39–2.25	1.75	1.38–2.23	0.81	0.62–1.06	0.82	0.63–1.07	0.62	0.48–0.79	0.62	0.49–0.80
10th grade	1.80	1.35–2.40	1.78	1.33–2.38	1.29	1.00–1.65	1.28	0.99–1.64	0.83	0.63–1.08	0.83	0.64–1.09	0.95	0.73–1.23	0.95	0.73–1.24
**Lockdown COVID-19 pandemic**																
Pre pandemic 2020	Ref		Ref		Ref		Ref		Ref		Ref		Ref		Ref	
lockdown	1.29	1.03–1.62	1.18	0.66–2.13	1.20	0.98–1.46	1.16	0.69–1.95	0.64	0.51–0.81	1.07	0.57–2.0	0.46	0.38–0.57	0.52	0.31–0.87
**Family SEP**																
High	Ref		Ref		Ref		Ref		Ref		Ref		Ref		Ref	
Medium	1.47	1.15–1.87	1.93	1.34–2.78	1.43	1.15–1.77	1.90	1.39–2.59	0.77	0.60–0.98	0.85	0.62–1.17	0.61	0.48–0.76	0.48	0.33–0.69
Low	1.66	1.20–2.30	1.97	1.30–3.00	1.90	1.43–2.53	2.20	1.53–3.16	0.80	0.58–1.12	0.70	0.47–1.05	0.47	0.35–0.64	0.40	0.27–0.60
**Interactions SEP** **×** **lockdown**																
Low^*^ lockdown			Ref				Ref				Ref				Ref	
Medium^*^ lockdown			0.60	0.37–0.98			0.58	0.38–0.90			0.78	0.48–1.30			1.47	0.93–2.34
High^*^ lockdown			0.71	0.36–1.39			0.75	0.41–1.36			1.63	0.81–3.27			1.37	0.74–2.54

* *Adjusted for school grade*.

## Results

### Mental Well-Being Among Adolescents Before and During the Lockdown Spring 2020

The first aim concerns the changes in mental well-being among adolescents prior to the pandemic (T1) and lockdown (T2) in spring 2020. First, the prevalence of students reporting high levels of “quality of life” and “life satisfaction” decreased significantly from T1 to T2 for both boys and girls. Table 1 shows that the percentage of boys reporting high levels of quality of life and life satisfaction decreased from 23 to 16 and 82 to 69% (*p* < 0.001), respectively. The corresponding decreases from T1 to T2 among girls are 23 to 16 and 82 to 69% (*p* < 0.001), respectively.

Furthermore, the prevalence of adolescents reporting high levels of depressive symptoms slightly increased from T1 to T2, although these increases were not statistically significant for either boys or girls. The results show, however, an increase of adolescents reporting high level of complaints in single items included in the depressive scale. For girls particularly, two items stand out; “*had sleep problems*” and “*felt unhappy, sad, or depressed*.” For boys, we only observed an increase in the proportion reporting high symptoms complaints related to the item “*felt hopeless about the future*.”

Turning to our parametric estimations, Tables 2, 3 show the associations between the four mental well-being outcomes (i.e., depressive symptoms, loneliness, quality of life, and life satisfaction) and lockdown adjusted for school grade level and family SEP in boys and girls, respectively. In girls we found that lockdown was associated with higher odds for reporting high depressive symptoms (OR = 1.29, 1.03–1.62). We did not find any association between the lockdown and self-reported loneliness in neither boys nor girls. The lockdown was negatively associated with quality of life and life satisfaction in both boys (OR = 0.65, 0.54–0.79; OR = 0.37, 0.28–0.49) and girls (OR = 0.64, 0.51–0.81; OR = 0.46, 0.38–1.57). In other words, the odds of reporting high life satisfaction were about 60% lower in boys and girls during the lockdown compared to pre-pandemic levels.

### Sociodemographic Inequalities in Adolescents' Mental Well-Being, Before and During Lockdown

To address our second research aim related to inequalities in student's mental well-being and quality of life between genders and socioeconomic groups, and whether the potential effects of these variables have changed during the pandemic, several logistic regression models were conducted. The results for each of the potential moderating factors examined are presented below. To aid interpretation, the statistical results of all analyses are described below and summarized in Table 2 (boys) and Table 3 (girls), Supplementary Table 1, and Figures 1, 2.

### Gender

Our parametric analyses (Supplementary Table 1) indicate that girls were more likely than boys to have high levels of depressive symptoms (OR = 3.05, 95% CI: 2.47–3.73) and loneliness (OR = 2.50, 95% CI: 2.12–2.95). Girls were less likely to report high self-reported quality of life (OR = 0.38, 95% CI: 0.33–0.44) and life satisfaction (OR = 0.48, 95% CI: 0.41–0.57).

Our moderating analyses (interaction models in Supplementary Table 1) show that the interaction term with the gender and lockdown was not statistically significant for any of the measures of mental well-being, indicating that the observed gender differences have not changed during the lockdown. Among boys and girls, the probability of high depressive symptoms and loneliness were higher in 9th and 10th grade students compared to students in 8th grade. We did not find similar patterns related to quality of life and life satisfaction. However, among 9th grade girls we found lower odds for high life satisfaction compared to girls at the 8th school grade level.

### Family SEP

Low family SEP is associated with higher odds of high depressive symptoms and loneliness in boys (OR = 2.33, *p* < 0.001; OR = 1.43, and *p* = 0.058) and girls (OR = 1.66, *p* < 0.01; OR = 1.90, and *p* < 0.001) compared with their high SEP pairs. Our analysis also suggests that high SEP adolescents were more likely to report high life satisfaction. High SEP boys and girls had more than twice the odds for reporting high life satisfaction compared with their low SEP peers. We did not find any inequalities between socioeconomic groups in quality of life among boys. Medium SEP girls had lower odds for reporting high quality of life (OR = 0.77, 95% CI: 0.60–0.98) compared with their high SEP counterparts.

Our moderating analyses (the interaction models shown in Tables 2, 3) do not provide support for any substantial changes in SEP inequalities in either boys or girls between T1 and T2. That is, the relative difference in depressive symptoms, loneliness, quality of life, or life satisfaction between socioeconomic groups did not change statistically significant between T1 and T2. However, we found the differences in the share of medium and high SEP girls reporting high depressive symptoms and loneliness narrowed between T1 and T2, as high SEP girls reported more problems.

Figures 1, 2 summarize supplementary analyses based on data in the period between 2014 and T2. The results of the parametric analyses suggest rising rates of high depressive symptoms and loneliness across socioeconomic groups and in both genders. Further, inequalities between the socioeconomic groups in successive surveys increased. The increase in prevalence of high depressive symptoms across surveys was lower in high SEP boys than in their low SEP peers (OR = 0.89, 95% CI: 0.80–0.99) during the study period. Girls showed a similar trend, but the increase was not statistically significant at 0.05 level (OR = 0.94, 95% CI: 0.88–1.01). Our analyses do not indicate that these SEP inequalities changed in any direction between T1 and T2; rather they seem to illustrate an ongoing trend of rising inequalities at least since 2014.

## Discussion

### Key Findings

The present study examines the impact of the societal lockdown during the first wave of the COVID-19 pandemic on the mental well-being of Norwegian adolescents. It also examines how gender and family SEP relate to adolescents' mental well-being and whether this potential association changed in comparison to the pre-pandemic situation. The results of this study suggest a significant decrease in quality of life and life satisfaction in both girls and boys during lockdown. For girls only, lockdown was associated with higher odds for reporting high depressive symptoms. As expected, we found distinct socioeconomic inequalities, with rising rates of psychological distress among the least privileged socioeconomic groups. However, our trend analyses provided no evidence that the socioeconomic inequalities in psychological distress (according to prevalence of high depressive symptoms or loneliness) changed between the pre-pandemic and lockdown periods.

### Rising Psychological Distress in the Adolescents During the First Wave of the COVID-19 Pandemic

The COVID-19 pandemic has significantly affected the lives of millions of children and adolescents around the world. Starting from the initial phase of the pandemic, children, adolescents, and their families have experienced a prolonged and collective stress related to myriad social changes. Norwegian adolescents have experienced at least one period in which they received home-based education, their regular leisure activities were put on hold, their physical and social contacts with friends and extended family decreased significantly, and they have spent more time with immediate family at home. All these factors can affect mental well-being in adolescence.

Although the prevalence of adolescents reporting high levels of depressive symptoms slightly increased during lockdown compared to the pre-pandemic situation, this finding was not statistically significant. During this period of time, there were many uncertainties associated with COVID-19. For example, it was not known for sure how the virus was transmitted or how deadly it was. This created many concerns for loved ones, especially those with conditions that were identified as risk factors early on. The economic implications and financial pressure created by the pandemic and the lockdown also affected some families. Significant stressors such as unemployment, income decline, and unmanageable debts typically harm the well-being of parents, influencing parent-child relationships and increasing children's risk of mental health problems (49). Evidence also suggests an increased incidence of domestic violence and intimate partner violence during this period (23).

All the stressors that increased in March 2020 are associated with considerable harms to young people's health and well-being, as well as their educational outcomes—which in turn affect health and socioeconomic conditions later in life (50–52). A recent review article identified high rates of anxiety, depression, and post-traumatic symptoms during the pandemic (21). Children and adolescents or parents with pre-existing mental health problems appear to be at the highest risk (18). Individual psychological effects of the pandemic are, in addition to individual variation, rooted in myriad societal changes at multiple layers of influence; community, family, and interpersonal (53).

However, the lockdown might have provided opportunities for adolescents, and their families. For example, some might have benefitted from spending more time together during lockdown as family members were brought closer together and experienced a sense of belonging and social support. In addition, lack of stressors from out-of-home leisure activities and private- or business-related arrangements during this time might have brought ease into family life. Moreover, mastering the pandemic related challenges together as a team may have strengthened the sense of community and cohesion among the family members.

Nonetheless, there is no doubt that lack of school services and regular leisure activities can increase adolescents' risk of loneliness and social isolation, both of which contribute to poor mental and physical health (9). At the community and inter-personal levels, adolescents had limited access to basic services such as schools, medical services, and leisure activities. Losing daily school routines meant losing a main source of normal daily rhythms and social cohesion with peers. During the first wave of the pandemic use of public playgrounds and participation in social group activities was prohibited. In addition, social relations was limited to immediate family members, depriving young people of the peer connections they normally rely on heavily for emotional support and social development (1, 2).

In a recent Norwegian study, (54) found that lack of physical contact with friends was associated with both depression and loneliness among adolescents during the pandemic. On average girls were lonelier than boys were, and they reported a higher level of depression symptoms. When asked about their biggest challenge, 20% indicated reduced social contact, isolation, and loneliness. Other studies showed similar findings (2, 28, 55, 56). It is thus unexpected that we did not find an increase in the proportion of adolescents reporting high levels of loneliness during the pandemic. One possible reason is that this study was conducted only 2 months into the pandemic, when people were still optimistic about the societal lockdown and the related severe social restrictions. Longitudinal follow-up studies are needed to explore whether self-perceived loneliness among different subgroups of the population have changed over the past year. Severe social restrictions are difficult to follow and may have detrimental effects for physical and mental health over time.

In line with our results, another study found that the adolescents in Norway reported a significant decrease in high self-reported life satisfaction (33). They also found that concerns about illness and infection were associated with lower life satisfaction scores. Two Nordic studies found that the high life satisfaction-scores were associated with less stressful everyday life with fewer academic demands, less social pressure, minimal difficult conditions at school, bullying or other type of conflicts, those with a small social network (57, 58). In addition, a Norwegian study found that children who managed better the period with home-based education, reported fewer somatic and cognitive problems (59).

Taken together, the complex interplay between risks and opportunities at different levels of society affects the psychological effects of the pandemic in family life and in the individual adolescents. Preexisting vulnerabilities and characteristics, within the individual adolescents and their respective families, significantly influence this complex interplay. The long-term effects of COVID-19 pandemic will, in other words, be highly individual and vary greatly in the population.

### Sociodemographic Variation in Adolescent's Mental Well-Being During the First Wave of the COVID-19 Pandemic

According to Van Lancker and Parolin (60) the COVID-19 pandemic is likely to amplify inequalities related to SEP and differences related to pre-existing vulnerabilities. In line with previous findings (11, 13, 15, 16), we found higher rates of high quality of life and life satisfaction among the most advantaged socioeconomic group, and higher rates of depressive symptoms and loneliness among the least advantaged. However, we did not find any proof of that these well-known inequalities have changed substantially during the lockdown. Notably, we found the relative proportion between medium and high-SEP girls reporting high level of loneliness was lower during the lockdown than pre-pandemic levels in spring 2020. The current literature concerning variations in psychological effects of the COVID-19 pandemic is conflicting (23, 33, 61). According to a narrative review by Fegert et al. (23), there are several indicators that socioeconomically disadvantaged children and adolescents are at highest risk for COVID-19 associated mental health effects. In many cases disadvantageous circumstances in one context often amplify adverse conditions in other contexts (62). Factors associated with the parents and family environment are, along with biological factors, the most important mediating variables between SEP and young people's mental well-being. Researchers suggest the reasons for this lie in the parents' psychological well-being and their resulting childrearing practices (63–65) as well as children's own material deprivation (66, 67). For instance, financial losses due to layoff or job loss will cause rising economic pressure on poor families. Previous recessions have exacerbated levels of child poverty, with long-lasting consequences for children's health, well-being, and learning outcomes (61). Moreover, there may be increasing inequalities between socioeconomic groups in parental support for home schooling and leisure activities (68). While learning might continue unimpeded for the adolescents from resourceful households, adolescents from households with fewer resources are likely to struggle more to complete homework and online courses because of their lack of resources.

In contrast, a Norwegian study by Von Soest et al. (33) found that the socioeconomic inequalities in adolescents' life satisfaction decreased during the lockdown. That is, societal lockdown seemed to affect life satisfaction in high SEP adolescents more negatively than their low SEP peers. One explanation is that high SEP youth participate to a greater extent in organized leisure activities, and they experienced the absence of such activities as a greater loss. This may also explain the reduction in the relative difference between medium and high SEP girls in self-reported loneliness during lockdown compared with pre-pandemic levels in our study.

Our findings of higher levels of depressive symptoms and loneliness in girls compared to boys are consistent with findings from other studies and national health reports (15, 16, 69, 70). Furthermore, our study suggests that girls are less likely to report high quality of life and life satisfaction. Girls have higher expectations in key life areas—such as education, sport and leisure activities and appearance (69, 71). According to Hankin et al. (72), girls are more socio-emotionally attentive than boys, and negative cognition style and ruminating may leave girls being more prone to mental health complaints, especially depressive symptoms. Notably, a gender differences in depressive symptoms increased during lockdown compared to the pre-pandemic situation, with more girls reporting sleep problems or felt unhappy, sad, or depressed. The boys were worried about the future. This gender gap could be explained by the gender differences in the expectations in key life areas which were impacted due to COVID-19 restrictions.

In evaluating a range of research on the impact of COVID-19 on the pandemic, it is, important to consider the cultural context and time of data collection. Similar to our study, most studies have examined only the acute impacts of lockdown on mental well-being and not the long-term effects as they were performed in the initial phase or during the first wave of the pandemic when the psychological effects are still limited. It is also important to consider the Norwegian welfare state when extrapolating from the current study to other countries. The Norwegian Government has introduced significant measures during the pandemic to secure jobs, help businesses and people, and strengthen health services. Consequently, the effects of the pandemic may be modified in the Norwegian population compared to other countries. As of this writing the pandemic is still ongoing and restrictions over time may reinforce already established SEP differences. There are many indications that the crisis will hit the least privileged group of the population the most (10, 59, 60). We have not seen the long-term effects of the pandemic yet and there is a need for longitudinal studies monitoring mental well-being over time in different subgroups of the population.

### Strength and Limitations

A main strength of this study is the use of a rich cross-sectional dataset based on repeated national surveys, which allowed us to explore potential psychological effects over a longer period and across socioeconomic groups of the study population. Furthermore, family SEP and adolescents' symptoms of depression were measured in a standardized manner by using validated measures (38, 39, 45). However, the cross-sectional nature of the study does not allow us to draw conclusions on cause-effect relationships. Longitudinal studies and with representative samples will be crucial for further understanding of the real psychological consequences among adolescents of the COVID-19 pandemics. Future studies should also explore possible mediating variables related to parents or family environment as well as the individual school and neighborhood/municipality of residence, as the consequences of restrictions may vary considerably. In Norway, the restriction has, to some extent, been place-dependent, as different municipalities responded to local outbreaks. A second limitation is that the municipalities of residence of participants included in the T1 and T2 sample may vary although they all lived in the same county. The socio-demographic distribution of the population of Norwegian municipalities varies and may have produced different results. Notably, the share of adolescents with a low family SEP was statistically lower in the T1 compared to the T2 sample. Third, our outcome variables are self-reported which introduce a risk of measurement or misclassification bias. Fourth, the reliability of the loneliness, quality of life and life satisfaction measures is uncertain and use of exclusively validated instruments would have strengthened the study findings. Fifth and finally, Ungdata (T1) always takes place during school hours to ensure equal conditions for all participants. However, this was not the case with the T2 sample as some participants completed the survey at home while the others in the classroom at school. Answering the survey at school ensures that students can sit undisturbed for the allotted time (1 h). Being able to talk to others or to have family members around might have affected the respondents.

## Conclusions

Adolescents are vulnerable and require careful consideration by their caregivers and healthcare system adaptations to allow for mental health support despite the lockdown. The current study suggests declining quality of life and life satisfaction among Norwegian adolescent boys and girls when compared to pre-pandemic to lockdown levels. Only girls had higher odds for reporting high depressive symptoms during the lockdown. Among, the least privileged socioeconomic groups, rising rates of psychological stress were identified. We found no evidence of these inequalities increasing during the first wave of the pandemic, other than the ongoing trend of rising inequalities over time. However, it is important to consider that this study was conducted in the early stages of the pandemic. Thus, there is a need for longitudinal studies exploring the psychological effect of the pandemic among adolescents during re-opening and post-pandemic phases. Current literature suggests that the pandemic and the societal lockdown will hit the least privileged groups of the population the most. Social and health policy, public health, and further research should focus on the least privileged groups.

## Data Availability Statement

Norwegian Social research (NOVA) administers and maintains the Ungdata database. Data is freely available for research and educational purposes from the Norwegian centre for research data (NSD) upon application. Details about the application process can be found at: https://nsd.no/nsddata/serier/ungdata_eng.html. Norwegian legislation prohibits deposition of these data to open archives.

## Ethics Statement

Ethical review and approval was not required for the study on human participants in accordance with the local legislation and institutional requirements. Written informed consent to participate in this study was provided by the participants' legal guardian/next of kin.

## Author Contributions

AM, RKV, and LRN contributed substantially to the conceptualization and design of the study. AM performed and had primary responsibility for all data management, statistical analysis, interpretation of the results, and writing the manuscript. RKV contributed to the design, planned, executed, and collected data in one of the surveys, and assisted in writing and editing the manuscript. LRN and IS assisted with the interpretation of the results and editing of the manuscript. All authors read and approved the final manuscript and take responsibility for the integrity of the data analysis and the decision to submit this manuscript for publication.

## Funding

The funding for open access publication fees was received from SINTEF AS.

## Conflict of Interest

The authors declare that the research was conducted in the absence of any commercial or financial relationships that could be construed as a potential conflict of interest.

## Publisher's Note

All claims expressed in this article are solely those of the authors and do not necessarily represent those of their affiliated organizations, or those of the publisher, the editors and the reviewers. Any product that may be evaluated in this article, or claim that may be made by its manufacturer, is not guaranteed or endorsed by the publisher.
