# Supplementary material for: Impact of COVID-19 Pandemic Lockdown on Mental Well-Being of Norwegian Adolescents During the First Wave—Socioeconomic Position and Gender Differences
Source: Front Public Health. 2021 Sep 14;9:717747. doi: 10.3389/fpubh.2021.717747 (PMC8476849; doi:10.3389/fpubh.2021.717747)
Supplement: Supplementary file 1 [file Data_Sheet_1.PDF]

frontiers

Supplementary Material

Supplementary table 1: The impact of gender and lockdown during covid-19 pandemic*, and its interaction on the probability of high level of depressive symptoms, loneliness, quality of life and life satisfaction among adolescents in lower secondary education

	**High depressive symptoms**				**Loneliness**				**Quality of life**				**Life satisfaction**			
	Main effect		Interaction		Main effect		Interaction		Main effect		Interaction		Main effect		Interaction	
**Predictors**	OR	95% CI	OR	95% CI	OR	95% CI	OR	95% CI	OR	95% CI	OR	95% CI	OR	95% CI	OR	95% CI
School grade																
8th grade	Ref		Ref		Ref		Ref		Ref		Ref		Ref		Ref	
9th grade	1.81	1.42-2.30	1.81	1.42-2.30	1.87	1.54-2.28	1.87	1.54-2.28	0.90	0.76-1.06	0.90	0.76-1.06	0.68	0.56-0.83	0.69	0.56-0.84
10th grade	1.74	1.36-1.36	1.74	1.36-2.23	1.42	1.16-1.75	1.42	1.16-1.74	0.86	0.72-1.03	0.86	0.72-1.03	0.95	0.77-1.16	0.95	0.77-1.17
Gender																
Boys	Ref		Ref		Ref		Ref		Ref		Ref		Ref		Ref	
Girls	3.04	2.47-3.73	2.92	2.18-3.92	2.50	2.12-2.95	2.59	2.05-3.28	0.38	0.33-0.44	0.38	0.31-0.46	0.48	0.41-0.57	0.42	0.32-0.55
Lockdown covid-19 pandemic																
Pre pandemic 2020	Ref		Ref		Ref		Ref		Ref		Ref		Ref		Ref	
Lockdown	1.28	1.06-1.28	1.21	0.86-1.71	1.23	1.05-1.44	1.29	0.99-1.68	0.65	0.56-0.75	0.66	0.54-0.79	0.43	0.36-0.51	0.37	0.28-0.49
Family SEP																
Low	Ref		Ref		Ref		Ref		Ref		Ref		Ref		Ref	
Medium	0.82	0.63-1.07	0.82	0.63-1.07	0.80	0.64-1.01	0.80	0.64-1.01	1.04	0.83-1.29	1.04	0.83-1.29	1.34	1.06-1.69	1.34	1.06-1.69
High	0.54	0.42-0.71	0.54	0.42-0.71	0.58	0.47-0.73	0.58	0.47-0.73	1.19	0.97-1.46	1.19	0.97-1.46	2.10	1.67-2.64	2.10	1.67-2.64
**Interactions gender x Lockdown**																
Female* Lockdown			Ref				Ref				Ref				Ref	
Male*Lockdown			1.08	0.71-1.62			0.93	0.67-1.29			0.98	0.73-1.31			1.24	0.88-1.75

*adjusted for school grade and family SEP

frontiers
